# Predictive Modeling of 30-Day Emergency Hospital Transport of German Patients Using a Personal Emergency Response: Retrospective Study and Comparison with the United States

**DOI:** 10.2196/25121

**Published:** 2021-03-08

**Authors:** Jorn op den Buijs, Marten Pijl, Andreas Landgraf

**Affiliations:** 1 Philips Research Eindhoven Netherlands; 2 Philips DACH Hamburg Germany

**Keywords:** emergency hospital transport, predictive modeling, personal emergency response system, population health management, emergency transport, emergency response system, emergency response, health management

## Abstract

**Background:**

Predictive analytics based on data from remote monitoring of elderly via a personal emergency response system (PERS) in the United States can identify subscribers at high risk for emergency hospital transport. These risk predictions can subsequently be used to proactively target interventions and prevent avoidable, costly health care use. It is, however, unknown if PERS-based risk prediction with targeted interventions could also be applied in the German health care setting.

**Objective:**

The objectives were to develop and validate a predictive model of 30-day emergency hospital transport based on data from a German PERS provider and compare the model with our previously published predictive model developed on data from a US PERS provider.

**Methods:**

Retrospective data of 5805 subscribers to a German PERS service were used to develop and validate an extreme gradient boosting predictive model of 30-day hospital transport, including predictors derived from subscriber demographics, self-reported medical conditions, and a 2-year history of case data. Models were trained on 80% (4644/5805) of the data, and performance was evaluated on an independent test set of 20% (1161/5805). Results were compared with our previously published prediction model developed on a data set of PERS users in the United States.

**Results:**

German PERS subscribers were on average aged 83.6 years, with 64.0% (743/1161) females, with 65.4% (759/1161) reported 3 or more chronic conditions. A total of 1.4% (350/24,847) of subscribers had one or more emergency transports in 30 days in the test set, which was significantly lower compared with the US data set (2455/109,966, 2.2%). Performance of the predictive model of emergency hospital transport, as evaluated by area under the receiver operator characteristic curve (AUC), was 0.749 (95% CI 0.721-0.777), which was similar to the US prediction model (AUC=0.778 [95% CI 0.769-0.788]). The top 1% (12/1161) of predicted high-risk patients were 10.7 times more likely to experience an emergency hospital transport in 30 days than the overall German PERS population. This lift was comparable to a model lift of 11.9 obtained by the US predictive model.

**Conclusions:**

Despite differences in emergency care use, PERS-based collected subscriber data can be used to predict use outcomes in different international settings. These predictive analytic tools can be used by health care organizations to extend population health management into the home by identifying and delivering timelier targeted interventions to high-risk patients. This could lead to overall improved patient experience, higher quality of care, and more efficient resource use.

## Introduction

The German population is one of five super-aged societies, and its population aged 65 years and older is projected to grow to about 24 million in 2050—roughly one-third of the total German population [1]. As a result, increasing demands are placed on the health care system due to chronic diseases that are more common in the elderly [2]. Emergency care in Germany is chronically overloaded: the number of patients seen in the emergency department (ED) doubled between 2005 and 2015 to around 25 million per year [3]. Older multimorbid patients make up an average of 30% of the patients treated in German EDs [4]. The number of hospital admissions by individuals aged 65 years and older is more than 49,000 per 100,000 annually [5]. The country’s health care sector has begun to leverage digital technology and eHealth solutions as part of a broader effort to accommodate a healthier and more engaged older population. Smartification of a person’s home through connected technologies has the potential to alleviate the shortage of nursing staff, support the desire of many elderly people to stay at home longer, and reduce costs for municipalities and health care services [6,7].

**Figure 1:**
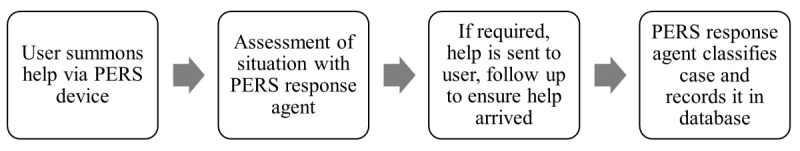
Overview of the personal emergency response system process and case data collection. PERS: personal emergency response system.

Such connected technologies include a personal emergency response system (PERS), which can help older adults get immediate assistance when a home-based incident occurs and where delayed response may result in preventable health care use such as ED visits [8]. The work described here builds on a previous study in which we used data obtained from a US PERS service to predict patients at high risk for imminent ambulance transport to the hospital [9]. PERS is a widely used wearable technology with a help button that is worn either as a bracelet or pendant. Subscribers may press the help button at any time to activate an in-home communication system that connects to a 24/7 response center. The response center associate may contact an informal responder (eg, a neighbor or family member) or emergency medical services (EMS) based on the subscriber’s specific situation, and follows up with the subscriber to confirm that help has arrived. The response center associate records notes from conversations with the subscribers in an electronic record and classifies the type, situation, and outcome of the case (Figure 1). In combination with user enrollment data, such as demographics, caregiver network, and medical condition data, these case data provide valuable information about subscriber status.

PERS services collect information while the subscriber is at home, including details such as timestamp, type, situation, and outcome of calls, that have either medical (eg, falls, respiratory issues, chest pain, or general pain) or social (eg, check-in calls) nature [10]. Such events may be indicative of decline in patient status, which may be captured earlier with PERS-based prediction models than with models based on only clinical data [11-14]. Previous efforts to predict health care use include predictive modeling of hospital readmission [11], repeat ED visits [12,13], and the use of specialized discharge services [14]. The LACE index uses 4 variables (length of stay [L], acuity of the admission [A], comorbidity of the patient [C], and emergency department use in the duration of 6 months before admission (E)) and was designed for the prediction of death or unplanned readmissions after hospitalization [15], achieving a predictive performance of AUC=0.68. HOSPITAL, a risk score for predicting 30-day potentially avoidable readmission, achieved a performance of AUC=0.72 as evaluated in 9 hospitals in 4 different countries [16]. Yet another study used 1-year retrospective electronic medical record data to predict 30-day ED revisits achieving AUC=0.70 in a prospective validation cohort [12].

As a next step, we designed and executed a 2-arm randomized control trial, which demonstrated that PERS-based risk prediction with targeted interventions could reduce health care use and costs [17,18]. In this study, a study nurse contacted high-risk subscribers, conducted additional triaging and, if deemed necessary, provided them with interventions including educational support, nurse home visits, or primary care physician referral. Based on the positive findings in the United States, we are investigating if a PERS-based risk prediction system with tailored interventions could also be applied in the German health care setting [19]. Similar to the US study, the German study requires a predictive model of risk of hospital transport in PERS users. Therefore, the objectives of this paper are to (1) develop and validate a predictive model of 30-day emergency hospital transport based on German PERS provider data and (2) compare the German and US models. It should be noted that various structural differences between the German and US PERS data prevented us from applying the US predictive model to the German data directly or using a transfer learning approach. Therefore, we opted to train a new prediction model on the German data.

## Methods

### Retrospective Data Set

The first study aim was to develop a 30-day predictive model of emergency hospital transport for a German PERS subscriber population. The initial retrospective data set used to develop the predictive model was extracted from the German PERS service provider ServiceCall AG [20]. It contained data from 8374 former PERS subscribers covering the period March 2006 through November 2018. Subscribers used a variety of PERS devices commercially available in Germany. At the time of study data collection, subscribers in the data set were deceased for at least 1 year to minimize impact on data privacy. This retrospective data study was approved by the Internal Committee for Biomedical Experiments of Philips (ICBE-2-24827).

The extract contained historical data including subscriber demographics such as gender, subscriber age at enrollment, and number of responders the subscriber had listed who could be contacted by the response center. The latter served as an indication of the size of the subscriber’s support network. In addition, the data set included self-reported medical conditions and medications provided by the subscriber at the time of enrollment.

Finally, the data set contained case data, which represent interactions with the response center such as incidents (where the subscriber requires assistance) or nonincidents such as test calls, false alarms, or technical issues. For interactions classified as incidents, a number of different situations were recorded (eg, subscriber has fallen), as well as a number of different follow-up actions, including contacting a friend or family member, having a conversation with the subscriber to jointly resolve the problem, or, in some cases, contact EMS.

### Inclusion and Exclusion Criteria

Subscribers were included in the analysis if they were active on the service at any time between January 1, 2012, and January 1, 2018. Subscribers were included in the analysis if they had a listed age between 18 and 100 years at the time of enrollment on the PERS service. Furthermore, subscribers were excluded if their contract end date predated the start date, presumably due to administrative error. Subscribers who did not have a unique identifier in the data set (ie, that shared a pseudonym with another subscriber) were also excluded. After applying inclusion and exclusion criteria, data from 5805 subscribers remained for analysis.

### Data Processing

The retrospective data set included a table consisting of subscriber data with a single row for each subscriber and a case data table with each row representing a single case. The tables were processed in the statistical programming software R (R Foundation for Statistical Computing).

The case data were characterized in terms of case types, case reasons, and case outcomes (Table 1). The case data for each subscriber were then aggregated by determining the frequency and recency of each of the case types, reasons, and outcomes. The frequency represents the number of a particular case that the subscriber has experienced, while the recency represents the time that has passed since the subscriber has experienced a particular case. Up to 2 years of historic case data were used to derive these features. The frequency and recency features of case data cases were then combined with subscriber demographics, support network, and self-reported medical conditions and medications from the subscriber data table. Tables were merged based on the pseudonymized subscriber IDs.

**Table 1:** Case types, reasons, and outcomes for which frequency and recency features are derived for input into the predictive model. Examples are given per category.

Classification example		Description
**Case type**		
	Incident	Case where the subscriber is in need of help
	Accidental	Subscriber accidentally pushed the help button
	Test	Test call by subscriber
**Case reason**		
	Fall	Subscriber fell
	Breathing problems	Subscriber has breathing problems
	Heart problems	Subscriber has heart problems
**Case outcome**		
	Nurse	Nurse visit
	Ambulance transport	Emergency medical services dispatched to bring subscriber to hospital
	No assistance required	Subscriber did not require further assistance

### Predictive Model Development

**Figure 2:**
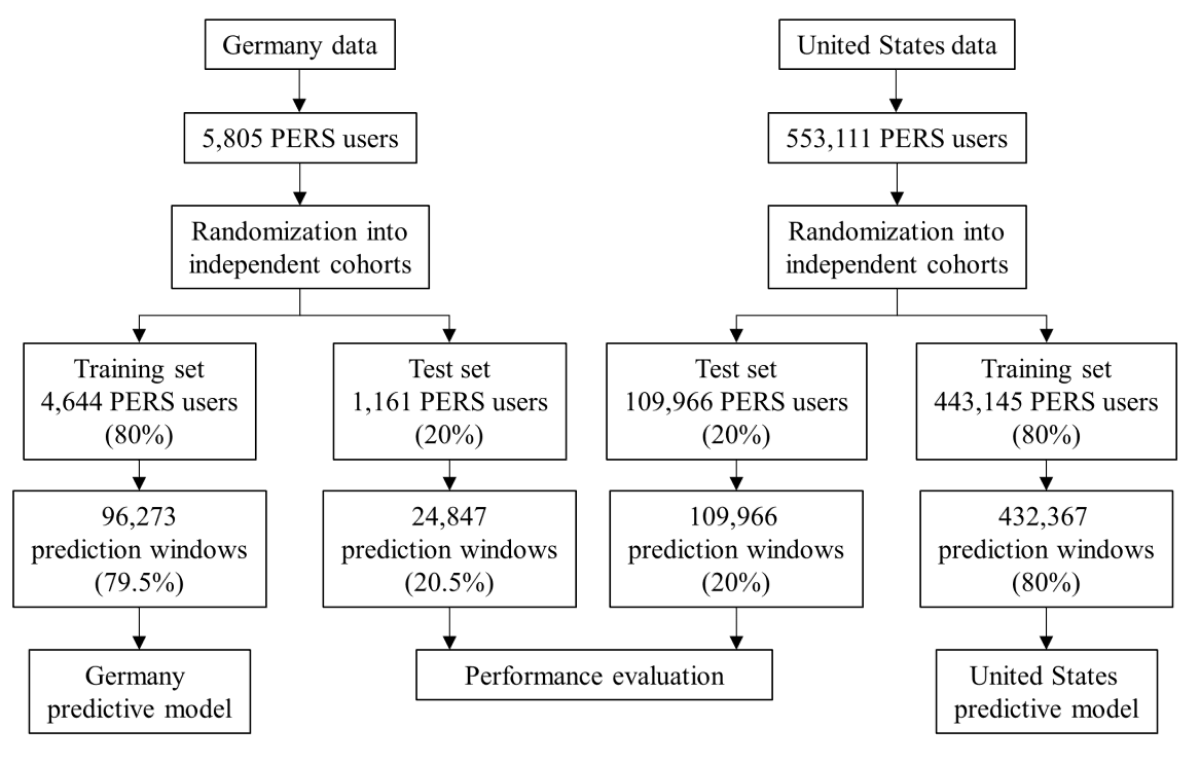
Overview of the study design to develop and evaluate the predictive models of emergency hospital transport. PERS: personal emergency response system.

**Figure 3:**
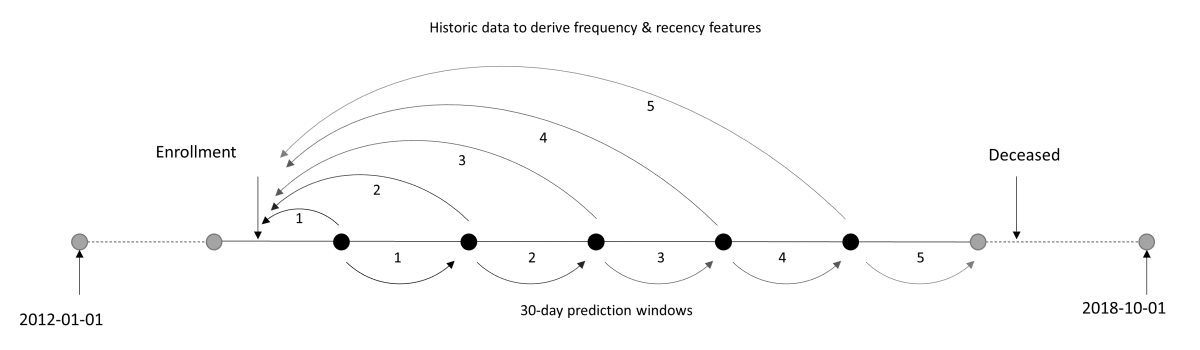
Schematic of using 30-day intervals to train and validate the prediction models.

The 5805 German PERS users were randomized into a training and test set in an 80:20 ratio (Figure 2). Originally, the US predictive model was trained using a 50:50 split of training and test set [9]. To eliminate the difference in training/test set ratio, the US predictive model was retrained on the US data set using an 80:20 split. Because the German data set was much smaller than the US data set, it was decided to create multiple prediction windows for each subscriber in order to use the data to the fullest extent possible. This was achieved by computing frequency and recency features and the dependent variable by splitting the data for each subscriber at multiple 30-day intervals (Figure 3). The dependent variable for the predictive model was determined as whether or not a subscriber had an event with the outcome “ambulance transport” in a 30-day prediction window (ie, the prediction was treated as a binary classification problem). The frequency and recency features for the predictive model were derived from the entire case data history prior to the 30-day window. It was ensured that training and test sets were independent (ie, data from a single subscriber were either in the training or the test set but not in both).

Based on the processed features, a predictive model for hospital transport was created using extreme gradient boosting, a variation of the boosted regression trees approach. Extreme gradient boosting is an ensemble approach where new models are added over a number of iterations in order to improve upon and correct the errors of the previous set of models. The models themselves take the form of small regression trees. In this study, XGBoost, an extreme gradient boosting algorithm implemented in R, was used since it has proven to perform well on nonlinear problems, including many high-ranking finishes in Kaggle data science competitions [21].

### Predictive Model Evaluation

Discriminatory accuracy of the predictive models was evaluated using area under the receiver operator characteristic curve (AUC), which indicates the probability of the predictive model ranking a randomly selected subscriber with 30-day emergency transport higher than a randomly selected subscriber without the event. Furthermore, the positive predictive value (PPV) indicates the percentage of subscribers having emergency transport in the group classified as positive (ie, having a 30-day emergency transport). The threshold for classifying subscribers as positive was varied using risk scores >90th, 95th and 99th percentile, such that 10%, 5%, and 1% of subscribers were classified as high risk, respectively. For these thresholds, the PPV, sensitivity, specificity, and accuracy were computed. Confidence intervals for performance metrics were derived using a stratified bootstrapping method with 1000 bootstrap replicates. The agreement between the predictions made by the model and the observed outcome was evaluated by plotting the average of the predicted probabilities and the observed percentage of users having 30-day emergency transport in deciles of the prediction score.

### Statistical Analysis

Differences between subscriber characteristics in the training and test sets of the German data and between both test sets of the German and US data were analyzed using Student *t* tests for age and chi-square tests for the categorical variables. Differences were considered to be statistically significant if *P*<.05.

## Results

### Subscriber Characteristics

Characteristics of subscribers in the training and test set of the Germany model are presented in Table 2 and compared with the test set used for the US predictive model. Data of 5805 unique PERS users were used in the Germany predictive model. A total of 4644 (80%) individuals were randomly selected for the training set and 1161 (20%) for the test set—training and test sets were mutually exclusive with regard to users. The US test set comprised 109,966 PERS users. Since the German data set was smaller, multiple prediction dates were considered by splitting the time range January 1, 2012, through January 1, 2018, into 73 equally spaced 30-day windows. This resulted in a training set containing 96,273 (79.5%) prediction dates and a test set with 24,847 (20.5%) prediction dates.

PERS users were on average aged 84.0 years in the German training set. Average age was slightly, but statistically significantly, younger in the test set (83.6 years). The average age in the US test set was statistically significantly lower at 81.2 years compared with the German test set. About two-thirds (2997/4644, 64.5%) of German PERS users were female, with no statistically significant difference between training and test sets. However, the US test set showed a significantly higher proportion of females (88,433/109,966, 80.4%).

In the German training set, more than half of users (2598/4644, 55.9%) were on the service 2 years or less, 23.2% (1079/4644) of users were 2 to 4 years on the service, and 20.8% (967/4644) of users were more than 4 years on the service. These percentages were not statistically significantly different in the test set. In the US test set, 44.4% (48,922/109,966) of users were less than 2 years on the service, which was significantly lower than in the Germany test set (630/1161, 54.3%). A similar percentage of US PERS users were 2 to 4 years on the service (26,193/109,966, 23.8%, vs 264/1161, 22.7%), while more users were 4 or more years on the service (34,851/109,966, 31.7%, vs 267/1161, 23.0%).

In the training set for the Germany predictive model, 94.3% (4397/4644) of users had at least one self-reported medical condition, with 35.7% (1657/4644) reporting 5 or more conditions. There were no statistically significant differences between the number of self-reported conditions in the training and test set for the Germany predictive model. In contrast, 77.3% (85,056/109,966) of users in the test set for the US predictive model self-reported one or more medical conditions, which was statistically significantly lower than in the test set for the Germany predictive model.

The prevalence of the dependent variable “emergency hospital transport in the next 30 days” was 1.6% (1506/96,273) in the training set and 1.4% (350/24,847) in the test set for the Germany predictive model. The latter was statistically significantly lower than the prevalence of the dependent variable in the test set of the US predictive model (2455/109,966, 2.2%).

**Table 2:** Subscriber characteristics and prevalence of the dependent variable in the training and test sets for the Germany predictive model compared with the previously published results of the US predictive model in the test set. *P* values are reported for differences between German test and training sets, and between US and German test sets.

Characteristics		Germany predictive model (this study)			US predictive model (from [9])	
		Training set	Test set	*P* value (test vs training Germany)	Test set	*P* value (US vs Germany test)
**General**						
	Prediction dates	Jan 1, 2012, to Jan 1, 2018	Jan 1, 2012, to Jan 1, 2018	—	Feb 1, 2014	—
	# of unique PERS^a^ users, n (%)	4644 (80)	116 (20)	—	109,966 (20)	—
	# of prediction windows, n (%)	96,273 (79.5)	24,847 (20.5)	—	109,966 (20)	—
	Age in years, mean (SD)	84.0 (8.2)	83.6 (8.3)	.001	81.1 (11.4)	<.001
	Female gender, n (%)	2997 (64.5)	743 (64.0)	.37	88,433 (80.4)	<.001
**Years on PERS service, n (%)**						
	0-2	2598 (55.9)	630 (54.3)	.32	48,922 (44.4)	<.001
	2-4	1079 (23.2)	264 (22.7)	.75	26,193 (23.8)	.41
	4 or more	967 (20.8)	267 (23.0)	.11	34,851 (31.7)	<.001
**Number of PERS self-reported medical conditions, n (%)**						
	None	265 (5.7)	73 (6.3)	.49	24,910 (22.6)	<.001
	1-2	1325 (28.5)	329 (28.3)	.93	26,515 (24.1)	<.001
	3-4	1397 (30.1)	370 (31.9)	.25	28,561 (26.0)	<.001
	5 or more	1657 (35.7)	389 (33.5)	.18	29,980 (27.3)	<.001
	30-day emergency hospital transport (% of prediction windows)	1506 (1.6)	350 (1.4)	.08	2455 (2.2)	<.001

^a^PERS: personal emergency response system.

### Predictive Model Evaluation

The performance of the Germany predictive model on the test set is detailed in Table 3 for various prediction score thresholds. AUC was 0.749 (95% CI 0.721-0.777) for emergency hospital transport in 30 days. This was slightly but not statistically significantly lower than the AUC for the US predictive model (0.778 [95% CI 0.769-0.788]), as 95% CIs were overlapping.

Positive predictive values for the Germany predictive model were low due to the low prevalence of 30-day emergency hospital transport, which was 1.4% (350/24,847) in the test set (Table 1). By increasing the prediction score threshold, PPV increased but at the expense of decreased sensitivity. At a prediction score threshold corresponding to the 90th percentile, the Germany predictive model identified 40.3% (95% CI 35.1%-45.4%) of the subscribers who had emergency transport in the 30 days following the prediction date (sensitivity); however, only 5.7% (95% CI 4.9%-6.4%) of flagged subscribers had emergency transport in the following 30 days (PPV) at this threshold. At thresholds corresponding to the 95th and 99th percentiles, the sensitivity dropped to 26.9% (95% CI 22.3%-31.1%) and 10.6% (95% CI 7.4%-14.0%), respectively, while the PPV increased to 7.5% (95% CI 6.3%-8.8%) and 15.0% (95% CI 10.7%-19.3%), respectively. When the threshold was set at the 99th percentile, the PPV was 10.7 times higher than the prevalence of 1.4%. This lift of the prediction model was similar for the US prediction model, namely 11.9.

The US predictive model demonstrated similar sensitivity and specificity values for the different thresholds. However, PPV was significantly higher across all thresholds compared with the Germany predictive model due to the higher prevalence of the target variable in the US data set.

**Table 3:** Performance of the Germany and US predictive models on the corresponding test sets, evaluated by positive predictive value, sensitivity, and specificity using the 90th, 95th, and 99th percentiles as a threshold and area under receiver operator characteristic curve.

Performance metric and threshold (percentile)		Germany predictive model (this study), % (95% CI)	US predictive model (adapted from [9]), % (95% CI)
**PPV^a^**			
	90%	5.7 (4.9-6.4)^b^	9.4 (8.9-9.8)
	95%	7.5 (6.3-8.8)^b^	13.6 (12.9-14.3)
	99%	15.0 (10.7-19.3)^b^	26.2 (23.7-28.5)
**Sensitivity**			
	90%	40.3 (35.1-45.4)	41.9 (40.0-43.9)
	95%	26.9 (22.3-31.1)	30.3 (28.6-32.0)
	99%	10.6 (7.4-14.0)	11.7 (10.5-13.0)
**Specificity**			
	90%	90.4 (90.1-90.8)	90.8 (90.6-91.0)
	95%	95.3 (95.1-95.6)	95.6 (95.6-95.7)
	99%	99.1 (99.0-99.2)	99.3 (99.2-99.3)
**AUC^c^**			
	—	0.749 (0.721-0.777)	0.778 (0.769-0.788)

^a^PPV: positive predictive value.

^b^Nonoverlapping 95% CI between Germany and US predictive models.

^c^AUC: area under the receiver operator characteristic curve.

**Figure 4:**
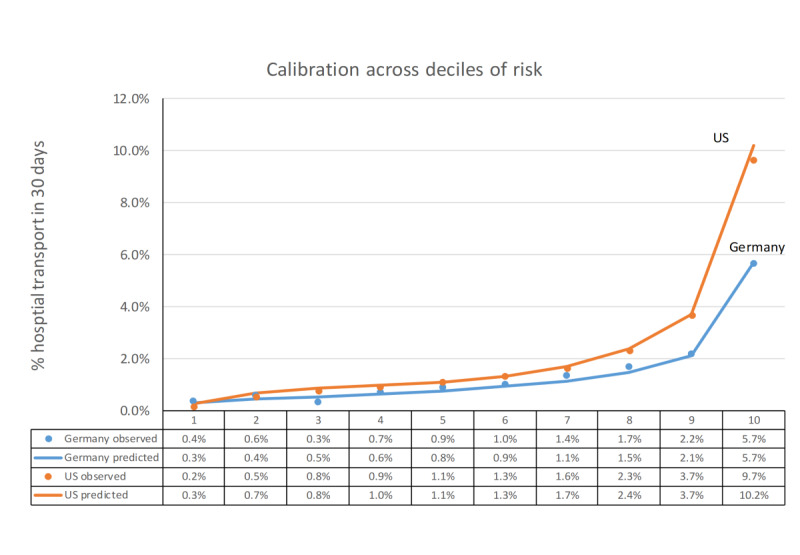
Observed percentage of subscribers needing 30-day emergency hospital transport versus model predicted probability across deciles of risk.

The predictive model produced for each subscriber a probability from 0% to 100% indicating the risk of having 30-day emergency hospital transport. The actually observed percentage of subscribers with 30-day emergency hospital transport and average predicted probabilities are presented in Figure 4 to indicate calibration across deciles of risk for both models. Each decile consists of 10% of the test set sorted by predicted probability. Probabilities increased from 0.4% in the lowest risk decile to 5.7% in the highest risk decile observed in the Germany test set and from 0.2% to 9.7% for the US test set. Both models were well calibrated with *R*^2^=.9935 and *R*^2^=.9992 for the Germany and US predictive models, respectively.

### Predictor Importance

The Germany predictive model of 30-day emergency hospital transport included 98 variables with nonzero values for the gain compared with 121 for the US predictive model. For each broad category of predictors, Table 4 provides the number of predictors and the gain. Here, gain is calculated by the XGBoost algorithm and represents a combined statistic of the information gain over all trees for a particular predictor. As such, gain represents a measure of the relative importance of individual predictors. Predictors from the case data form the most important predictor category for both predictive models, although percentage-wise, their contribution to the Germany predictive model was lower compared with the US predictive model (72.9% vs 87.7%, respectively). On the other hand, the relative importance of self-reported medical conditions (9.6% vs 3.7%, respectively) and other predictors (17.5% vs 8.7%, respectively) was higher in the Germany predictive model.

**Table 4:** Number of predictors and total gain per predictor category.

Predictor category	Number of predictors		Total gain, %	
	Germany	US	Germany	US
Case data-based predictors	48	62	72.9	87.7
Self-reported medical conditions	43	44	9.6	3.7
Other predictors	7	15	17.5	8.7
Total	98	121	100	100

**Figure 5:**
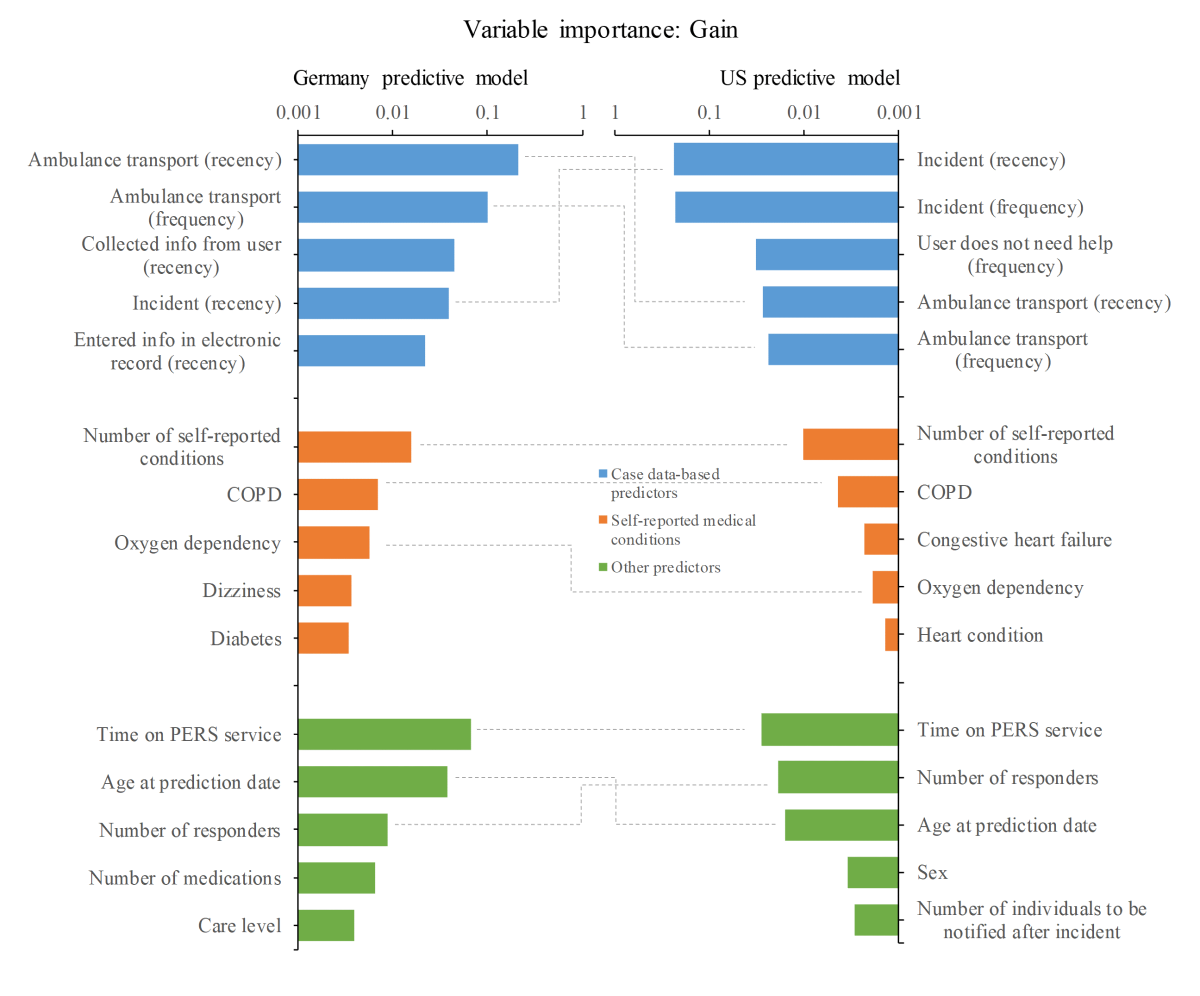
Predictor importance as measured by the gain of the 5 most important variables in 3 categories of predictors for both Germany and US predictive models. Predictor categories: case data, self-reported medical conditions, and other predictors. Dashed lines indicate features present in both predictive models. COPD: chronic obstructive pulmonary disease; PERS: personal emergency response system.

The 5 most important predictors for each category are shown in Figure 5 for both models. In each category, 3 out of 5 predictors were overlapping between the Germany and US predictive models. For case data–based predictors, these included recency and frequency of ambulance transport and recency of incidents. In the Germany predictive model, features based on the collection of further case information from the user and entering additional information into the electronic record were found among the important predictors. We expect that extracting features from these free text notes in the electronic record could lead to further model improvement; however, text notes were left out of the analysis due the risk of including privacy-sensitive information.

Furthermore, the number of self-reported medical conditions, chronic obstructive pulmonary disease, and oxygen dependency were among the 5 most important predictors in both models in the category self-reported medical conditions. Other important predictors that were shared by both models included time on the PERS service, age, and number of responders. It should be noted that in the Germany predictive model, number of medications and care level (Pflegestufe), a Germany-specific categorization of the level of (financial) support individuals need with activities of daily living, were among the most important other predictors, and this information was not available in the US PERS data.

## Discussion

### Principal Findings

In previous work, we have shown that PERS service data from US subscribers can be used to predict risk for emergency hospital transport [9]. This study is an extension of that work to determine the feasibility to develop such a prediction model on data from German PERS subscribers. Comparison of data from the German and US PERS providers shows that PERS users in both countries have, on a high level, similar characteristics—average age over 80 years, predominantly female, and more than half reporting on 3 or more medical conditions. On the other hand, Table 2 shows various subtle differences between the characteristics of the two populations, justifying the effort to develop a Germany-specific predictive model.

In the US and German PERS populations, the prevalence of hospital transport in 30 days among PERS users was significantly lower in the German PERS population (1.4% vs 2.2%, respectively). Health insurance is obligatory in Germany, and the health care systems covers all costs of both inpatient and outpatient treatment [22]. Compared with the United States, where patients often self-refer to the ED out of financial considerations [23], this is therefore less likely to play a role in Germany, which might explain the difference in prevalence of hospital transport. Nevertheless, the increasing number of ED visits that could have been prevented via treatment in the primary care setting is a growing issue in Germany.

Evaluation of the German prediction model of 30-day emergency hospital transport on a test set of data from different PERS users demonstrated that at-risk subscribers could be identified with discriminatory accuracy similar to the US prediction model (AUC=0.749 vs AUC=0.778). Furthermore, calibration across deciles indicated that the predicted probabilities for both the Germany and US prediction models closely matched with observed outcomes. Calibration refers to the agreement between observed outcomes and predictions (ie, if we predict a 10% risk of 30-day hospital transport, the observed frequency of hospital transport should be approximately 10 out of 100 subscribers with such a prediction [24]). Finally, analysis of variable importance indicated that predictors derived from the medical alert pattern data, including the frequency and recency of prior ambulance transports, were most predictive of future hospital transport in both the German and US prediction models. Similarly, Poole et al [25] found that the timing and frequency of prior ED use are the strongest predictors of future ED visits using a random forest model.

Our previous study on health care use in US PERS users indicates that 21% of hospital admissions are considered potentially avoidable [10]. A recent study on hospitalizations by German nursing home patients classified 27% as potentially avoidable [26]. Therefore, we believe that prediction of emergency transport risk in combination with appropriate interventions could potentially reduce health care use. Case managers and health professionals should integrate risk prediction of patients into their clinical workflows to obtain the clinical and financial benefits from predictive models, which requires a detailed guideline that clarifies how the algorithm will inform care [27]. In a recently completed randomized clinical trial, we developed workflows that integrate daily PERS-based risk of 30-day emergency hospital transport with care pathways [17], resulting in 49% fewer EMS encounters in the intervention group [18]. In a currently running prospective study in Germany [19], the predictive model described herein is used to predict subscribers risk for 30-day emergency transport, followed by a case manager assessment, and tailored interventions for high-risk subscribers. The number of patients who will ultimately benefit from a combination of prediction and intervention will depend on various factors including the population size and prevalence of emergency health care use, performance of the predictive model and risk threshold above which patients are considered to be high risk, and efficacy of the interventions provided to high-risk patients.

In our prospective study in Germany [19], the predicted risk scores drive proactive outreach—if the risk is above a certain threshold, the patient may be contacted by the case manager. Due to the low prevalence of hospital transport in the German PERS population, setting the value of the risk threshold is a trade-off between finding many true positive cases (ie, a high sensitivity) and reducing the number of false positives (ie, a high PPV), as shown in Table 2 and also reported by other predictive modeling studies of emergency health care use [12,28]. Despite this, our recent study in a US PERS population has demonstrated that health care use and cost can be reduced by combining risk prediction with preventive interventions [18].

### Limitations

This study had a few limitations. The PERS population is mostly older and primarily female, and the service is to a certain extent privately paid for by subscribers (ie, not fully covered by their health insurance). This may limit the generalizability of the study to older women who can afford the service. Furthermore, the predictive model may have been influenced by confounding of unobserved variables, including when and where users wear the PERS device [29].

Subscribers may have initiated emergency hospital transport outside of the PERS service, in which case there are no records in the PERS data, which may have affected predictive model development. As a mitigation measure, participants of our prospective study are instructed to use their emergency pendant for all incidents where they require help.

### Conclusions

This study showed that remotely collected subscriber data from a German PERS service can be used to predict 30-day hospital transport with similar discriminatory accuracy and calibration as our previously published prediction model developed on data from a US PERS population. Health care providers could potentially benefit from our validated predictive model by estimating the risk of 30-day emergency hospital transport for individual subscribers and target timely preventive interventions to high-risk subscribers. Due to a lower prevalence of emergency hospital transport in Germany compared with the United States, it needs further investigation if combining risk prediction with interventions will effectively reduce health care use. We are currently testing this hypothesis in a prospective study where risk predictions are combined with a stepped intervention pathway. This approach could lead to overall improved patient experience, higher quality of care, and more efficient resource use.

## Abbreviations

AUC: area under the receiver operator characteristic curve
ED: emergency department
EMS: emergency medical services
PERS: personal emergency response system
PPV: positive predictive value
